# Motor symptoms in genetic frontotemporal dementia: developing a new module for clinical rating scales

**DOI:** 10.1007/s00415-022-11442-y

**Published:** 2022-11-17

**Authors:** Kiran Samra, Amy M. MacDougall, Georgia Peakman, Arabella Bouzigues, Martina Bocchetta, David M. Cash, Caroline V. Greaves, Rhian S. Convery, John C. van Swieten, Lize Jiskoot, Harro Seelaar, Fermin Moreno, Raquel Sanchez-Valle, Robert Laforce, Caroline Graff, Mario Masellis, Carmela Tartaglia, James B. Rowe, Barbara Borroni, Elizabeth Finger, Matthis Synofzik, Daniela Galimberti, Rik Vandenberghe, Alexandre de Mendonça, Chris R. Butler, Alexander Gerhard, Simon Ducharme, Isabelle Le Ber, Pietro Tiraboschi, Isabel Santana, Florence Pasquier, Johannes Levin, Markus Otto, Sandro Sorbi, Jonathan D. Rohrer, Lucy L. Russell, the Genetic FTD Initiative (GENFI), Annabel Nelson, Martina Bocchetta, David Cash, David L. Thomas, Emily Todd, Hanya Benotmane, Jennifer Nicholas, Kiran Samra, Rachelle Shafei, Carolyn Timberlake, Thomas Cope, Timothy Rittman, Alberto Benussi, Enrico Premi, Roberto Gasparotti, Silvana Archetti, Stefano Gazzina, Valentina Cantoni, Andrea Arighi, Chiara Fenoglio, Giorgio Fumagalli, Vittoria Borracci, Giacomina Rossi, Giorgio Giaccone, Giuseppe Di Fede, Paola Caroppo, Pietro Tiraboschi, Sara Prioni, Veronica Redaelli, David Tang-Wai, Ekaterina Rogaeva, Miguel Castelo-Branco, Morris Freedman, Ron Keren, Sandra Black, Sara Mitchell, Christen Shoesmith, Robart Bartha, Rosa Rademakers, Jackie Poos, Janne M. Papma, Lucia Giannini, Rick van Minkelen, Yolande Pijnenburg, Benedetta Nacmias, Camilla Ferrari, Cristina Polito, Gemma Lombardi, Valentina Bessi, Michele Veldsman, Christin Andersson, Hakan Thonberg, Linn Öijerstedt, Vesna Jelic, Paul Thompson, Tobias Langheinrich, Albert Lladó, Anna Antonell, Jaume Olives, Mircea Balasa, Nuria Bargalló, Sergi Borrego-Ecija, Ana Verdelho, Carolina Maruta, Catarina B. Ferreira, Gabriel Miltenberger, Frederico Simões do Couto, Alazne Gabilondo, Ana Gorostidi, Jorge Villanua, Marta Cañada, Mikel Tainta, Miren Zulaica, Myriam Barandiaran, Patricia Alves, Benjamin Bender, Carlo Wilke, Lisa Graf, Annick Vogels, Mathieu Vandenbulcke, Philip Van Damme, Rose Bruffaerts, Koen Poesen, Pedro Rosa-Neto, Serge Gauthier, Agnès Camuzat, Alexis Brice, Anne Bertrand, Aurélie Funkiewiez, Daisy Rinaldi, Dario Saracino, Olivier Colliot, Sabrina Sayah, Catharina Prix, Elisabeth Wlasich, Olivia Wagemann, Sandra Loosli, Sonja Schönecker, Tobias Hoegen, Jolina Lombardi, Sarah Anderl-Straub, Adeline Rollin, Gregory Kuchcinski, Maxime Bertoux, Thibaud Lebouvier, Vincent Deramecourt, Beatriz Santiago, Diana Duro, Maria João Leitão, Maria Rosario Almeida, Miguel Tábuas-Pereira, Sónia Afonso

**Affiliations:** 1 Dementia Research Centre, Department of Neurodegenerative Disease, UCL Queen Square Institute of Neurology, Queen Square, London, WC1N 3BG UK; 2 Department of Medical Statistics, London School of Hygiene and Tropical Medicine, London, UK; 3 Department of Neurology, Erasmus Medical Centre, Rotterdam, Netherlands; 4 Cognitive Disorders Unit, Department of Neurology, Donostia Universitary Hospital, San Sebastian, Spain; 5 Neuroscience Area, Biodonostia Health Research Institute, Gipuzkoa, San Sebastian, Spain; 6 Alzheimer’s Disease and Other Cognitive Disorders Unit, Neurology Service, Hospital Clínic, Institut d’Investigacións Biomèdiques August Pi I Sunyer, University of Barcelona, Barcelona, Spain; 7 Clinique Interdisciplinaire de Mémoire, Département des Sciences Neurologiques, CHU de Québec, and Faculté de Médecine, Université Laval, Quebec City, Quebec Canada; 8 Center for Alzheimer Research, Division of Neurogeriatrics, Department of Neurobiology, Care Sciences and Society, Bioclinicum, Karolinska Institutet, Solna, Sweden; 9 Unit for Hereditary Dementias, Theme Aging, Karolinska University Hospital, Solna, Sweden; 10 Sunnybrook Health Sciences Centre, Sunnybrook Research Institute, University of Toronto, Toronto, Canada; 11 Tanz Centre for Research in Neurodegenerative Diseases, University of Toronto, Toronto, ON Canada; 12 Department of Clinical Neurosciences, University of Cambridge, Cambridge, UK; 13 Neurology Unit, Department of Clinical and Experimental Sciences, University of Brescia, Brescia, Italy; 14 Department of Clinical Neurological Sciences, University of Western Ontario, London, ON Canada; 15 Department of Neurodegenerative Diseases, Hertie-Institute for Clinical Brain Research and Center of Neurology, University of Tübingen, Tübingen, Germany; 16 Center for Neurodegenerative Diseases (DZNE), Tübingen, Germany; 17 Fondazione Ca’ Granda, IRCCS Ospedale Policlinico, Milan, Italy; 18 University of Milan, Centro Dino Ferrari, Milan, Italy; 19 Laboratory for Cognitive Neurology, Department of Neurosciences, KU Leuven, Leuven, Belgium; 20 Neurology Service, University Hospitals Leuven, Leuven, Belgium; 21 Leuven Brain Institute, KU Leuven, Leuven, Belgium; 22 Laboratory of Neurosciences, Institute of Molecular Medicine, Faculty of Medicine, University of Lisbon, Lisbon, Portugal; 23 Nuffield Department of Clinical Neurosciences, Medical Sciences Division, University of Oxford, Oxford, UK; 24 Department of Brain Sciences, Imperial College London, London, UK; 25 Division of Neuroscience and Experimental Psychology, Wolfson Molecular Imaging Centre, University of Manchester, Manchester, UK; 26 Departments of Geriatric Medicine and Nuclear Medicine, University of Duisburg-Essen, Essen, Germany; 27 Department of Psychiatry, McGill University Health Centre, McGill University, Montreal, QC Canada; 28 McConnell Brain Imaging Centre, Montreal Neurological Institute, McGill University, Montreal, QC Canada; 29 Paris Brain Institute-Institut du Cerveau-ICM, Inserm U1127, CNRS UMR 7225, AP-HP-Hôpital Pitié-Salpêtrière, Sorbonne Université, Paris, France; 30 Centre de référence des démences rares ou précoces, IM2A, Département de Neurologie, AP-HP-Hôpital Pitié-Salpêtrière, Paris, France; 31 Département de Neurologie, AP-HP-Hôpital Pitié-Salpêtrière, Paris, France; 32 Reference Network for Rare Neurological Diseases (ERN-RND), Paris, France; 33 Fondazione IRCCS Istituto Neurologico Carlo Besta, Milan, Italy; 34 Neurology Service, Faculty of Medicine, University Hospital of Coimbra (HUC), University of Coimbra, Coimbra, Portugal; 35 Center for Neuroscience and Cell Biology, Faculty of Medicine, University of Coimbra, Coimbra, Portugal; 36 Univ Lille, Lille, France; 37 Inserm 1172, Lille, France; 38 CHU, CNR-MAJ, Labex DistalzLiCEND Lille, Lille, France; 39 Department of Neurology, Ludwig-Maximilians Universität München, Munich, Germany; 40 German Center for Neurodegenerative Diseases (DZNE), Munich, Germany; 41 Munich Cluster of Systems Neurology (SyNergy), Munich, Germany; 42 Department of Neurology, University of Ulm, Ulm, Germany; 43 Department of Neurofarba, University of Florence, Florence, Italy; 44 IRCCS Fondazione Don Carlo Gnocchi, Florence, Italy

**Keywords:** Frontotemporal dementia, Genetics, Motor, Tau, Progranulin, *C9orf72*

## Abstract

**Objective:**

To investigate the optimal method of adding motor features to a clinical rating scale for frontotemporal dementia (FTD).

**Methods:**

Eight hundred and thirty-two participants from the international multicentre Genetic FTD Initiative (GENFI) study were recruited: 522 mutation carriers (with *C9orf72*, *GRN* and *MAPT* mutations) and 310 mutation-negative controls. A standardised clinical questionnaire was used to assess eight motor symptoms (dysarthria, dysphagia, tremor, slowness, weakness, gait disorder, falls and functional difficulties using hands). Frequency and severity of each motor symptom was assessed, and a principal component analysis (PCA) was performed to identify how the different motor symptoms loaded together. Finally, addition of a motor component to the CDR^®^ plus NACC FTLD was investigated (CDR^®^ plus NACC FTLD-M).

**Results:**

24.3% of mutation carriers had motor symptoms (31.7% *C9orf72*, 18.8% *GRN*, 19.3% *MAPT*) compared to 6.8% of controls. Slowness and gait disorder were the commonest in all genetic groups while tremor and falls were the least frequent. Symptom severity scores were similar to equivalent physical motor examination scores. PCA revealed that all motor symptoms loaded together so a single additional motor component was added to the CDR^®^ plus NACC FTLD to form the CDR^®^ plus NACC FTLD-M. Individual global scores were more severe with the CDR^®^ plus NACC FTLD-M, and no patients with a clinically diagnosed motor disorder (ALS/FTD-ALS or parkinsonism) were classified anymore as asymptomatic (unlike the CDR^®^ plus NACC FTLD alone).

**Conclusions:**

Motor features are present in mutation carriers at all disease stages across all three genetic groups. Inclusion of motor symptoms in a rating scale that can be used in future clinical trials will not only ensure a more accurate severity measure is recorded but that a wider spectrum of FTD phenotypes can be included in the same trial.

**Supplementary Information:**

The online version contains supplementary material available at 10.1007/s00415-022-11442-y.

## Introduction

Frontotemporal dementia (FTD) is a neurodegenerative disorder that can present with a wide spectrum of phenotypes including behavioural, language and motor symptoms. It is often a sporadic condition but in around a third of individuals it is inherited, with the main autosomal dominant genetic mutations being found in progranulin (*GRN*), microtubule-associated protein tau (*MAPT*) and chromosome 9 open reading frame 72 (*C9orf72*) [1, 2]. Whilst there are currently no disease-modifying therapies for FTD, trials are now underway, and efforts are being made to develop robust outcome measures. However, clinical rating scales have so far focused only on changes in behaviour and language, with the exclusion of motor features.

Importantly, and highlighting the need to include such symptoms in outcome measures, motor features can develop not only as the disease progresses in people whose presenting phenotype is behavioural (behavioural variant FTD, bvFTD) or linguistic (primary progressive aphasia, PPA), but also as part of a primary phenotype. The main motor syndromes in FTD are atypical parkinsonian disorders, including progressive supranuclear palsy (PSP) and corticobasal syndrome (CBS) [3, 4], and motor neurone disease (MND), also known as amyotrophic lateral sclerosis, ALS [5]. Classical PSP (Richardson’s syndrome) involves an early vertical supranuclear gaze palsy with an akinetic-rigid syndrome and subsequent falls [6], although it is now well-recognised that PSP can overlap with other syndromes including bvFTD and PPA [7, 8], [9]. CBS is characterised by progressive asymmetric rigidity and apraxia with additional features including alien limb syndrome, cortical sensory loss and myoclonus [10, 11], and has also been shown to overlap with both bvFTD and PPA [12, 13]. ALS causes progressive weakness, muscle atrophy and fasciculations [14], with around 15–20% of people developing FTD (usually bvFTD rather than PPA, and referred to as FTD-MND or FTD-ALS) and a further 30–40% developing cognitive impairment not meeting criteria for FTD [15–19].

In genetic FTD, motor features are seen in all three of the major genetic causes. *C9orf72* expansions are the commonest cause of genetic ALS, with the motor syndrome occurring alone or in combination with FTD (Devenney et al., 2015). However, some studies have also shown that *C9orf72* mutation carriers can develop atypical parkinsonism [20, 21], although it is uncommon for this to be the initial presentation [22]. ALS is extremely rare in those with *GRN* or *MAPT* mutations [22] but a substantial minority can present with parkinsonism. In ~ 5% of *GRN* mutation carriers CBS is the presenting syndrome, although it can also develop subsequently in other patients whose initial syndrome is bvFTD or PPA [22–25]. Similarly, *MAPT* mutation carriers can also present with CBS (in 2% in one study, Moore et al., 2020) as well with PSP (4%) or with a parkinsonian syndrome that resembles idiopathic Parkinson’s disease (PD, up to 5%) [26–30].

Whilst motor features exist in individuals with FTD with and without a primary motor diagnosis they are not yet included in any of the main rating scales currently used for FTD, e.g. the CDR^®^ plus NACC FTLD [31] or FTD Rating Scale [32]. Inclusion of this domain will be important in any comprehensive scale of the FTD spectrum, as motor deficits and functional impairment are closely aligned and may impact quality of life [33–35].

This study aims to understand the frequency and severity of motor features in genetic FTD using data from the international multicentre Genetic FTD Initiative (GENFI). Through this analysis, the study aims to investigate how best to incorporate motor symptoms into any FTD scale. This will be important for future clinical trials, allowing more accurate measurement of progression and treatment response, as well as offering insight into the impact of motor features on the lives of patients living with FTD and their caregivers.

### Methods

#### Participants

Participants were recruited from the fifth data freeze of the GENFI study between 20 January 2012 and 30 May 2019, including sites in the UK, Canada, Belgium, Germany, Italy, Netherlands, Portugal, Spain, and Sweden. All aspects of the study were approved by local ethics committees, and written informed consent obtained from all participants.

The standardised GENFI clinical assessment includes a history, examination, cognitive assessment, and the CDR^®^ plus NACC FTLD rating scale [31]. Mutation carriers were classified into asymptomatic, prodromal, or symptomatic if they scored 0, 0.5 or ≥ 1, respectively, on the CDR^®^ plus NACC FTLD global score.

All mutation carriers with baseline clinical data were included: 522 in total, consisting of 221 *C9orf72*, 213 *GRN*, and 88 *MAPT* mutation carriers. Within this group 291 (56%) were asymptomatic, 82 (16%) were prodromal, and 149 (29%) were fully symptomatic according to CDR^®^ plus NACC FTLD global score (Table 1). Symptomatic status was also assessed separately by a clinician, judging people to have a clinical diagnosis based on consensus criteria: from this assessment 110 had bvFTD [36], 26 had PPA (of whom 16 had nonfluent variant, one had semantic variant and nine had PPA not meeting criteria for any of the core syndromes) [37], 17 had ALS or FTD-ALS [19] and five had a parkinsonian syndrome [9, 38]. The control group consisted of at-risk family members who were mutation negative (and had a CDR^®^ plus NACC FTLD global score of 0 or 0.5): 310 in total. Demographics of the groups are shown in Table 1.

**Table 1:** Demographics, clinical scores and frequency of motor symptoms for all mutation carriers (*C9orf72, GRN, MAPT*) and healthy controls

	Controls	All mutation carriers				*C9orf72*				*GRN*				*MAPT*			
CDR® plus NACC FTLD		All	0	0.5	1 +	All	0	0.5	1 +	All	0	0.5	1 +	All	0	0.5	1 +
Number of participants	310	522	291	82	149	221	112	37	72	213	130	31	52	88	49	14	25
% Male	44	44	38	41	58	49	41	41	65	39	35	48	46	45	41	29	64
Age (years)	46.0 (12.7)	50.1 (13.7)	44.2 (11.9)	49.7 (12.3)	62.0 (9.2)	51.2 (13.6)	44.5 (11.7)	49.4 (11.2)	62.7 (9.3)	51.0 (13.6)	45.8 (12.2)	51.8 (13.2)	63.5 (7.7)	45.3 (13.1)	39.2 (10.4)	45.7 (12.6)	57.0 (10.1)
Education (years)	14.5 (3.3)	13.9 (3.4)	14.5 (3.2)	13.9 (3.1)	12.8 (3.7)	13.9 (3.2)	14.4 (3.0)	14.1 (2.6)	13.1 (3.7)	13.9 (3.7)	14.7 (3.4)	14.0 (4.0)	11.9 (3.5)	14.1 (3.3)	14.4 (3.3)	13.5 (2.4)	13.6 (3.8)
MMSE	29.3 (1.0)	27.1 (5.3)	29.3 (1.0)	28.5 (2.2)	21.9 (7.6)	27.2 (4.7)	29.1 (1.2)	28.5 (2.1)	23.4 (6.6)	26.9 (6.0)	29.4 (0.9)	28.5 (2.4)	19.3 (8.4)	27.4 (5.1)	29.5 (0.8)	28.2 (2.3)	22.8 (7.7)
CDR^®^ plus NACC FTLD Global score	0.1 (0.2)	0.6 (1.0)	0.0 (0.0)	0.5 (0.0)	2.0 (0.8)	0.8 (1.0)	0.0 (0.0)	0.5 (0.0)	2.1 (0.8)	0.5 (0.9)	0.0 (0.0)	0.5 (0.0)	1.9 (0.8)	0.6 (0.9)	0.0 (0.0)	0.5 (0.0)	1.9 (0.8)
CDR^®^ plus NACC FTLD Sum of Boxes	0.2 (0.4)	3.1 (5.5)	0.0 (0.0)	1.1 (0.8)	10.3 (5.8)	3.8 (6.0)	0.0 (0.0)	1.1 (0.8)	10.9 (5.7)	2.5 (5.1)	0.0 (0.0)	1.0 (0.8)	9.8 (6.0)	2.9 (5.3)	0.0 (0.0)	1.1 (0.8)	9.7 (5.8)
Motor symptoms %	6.8	24.3	5.5	20.7	63.1	31.7	7.1	32.4	69.4	18.8	4.6	9.7	59.6	19.3	4.1	14.3	52.0
Motor diagnosis %	0.0	4.2	1.4	6.1	8.7	7.7	2.7	13.5	12.5	1.4	0.0	0.0	5.8	2.3	2.0	0.0	4.0

Age, education, Mini-Mental State Examination (MMSE) and clinical rating scale scores are shown as mean (standard deviation)

#### Motor features

Motor symptoms were investigated within the history assessment using a standardised questionnaire developed for the GENFI study, and consisting of eight motor symptoms, dysarthria, dysphagia, tremor, slowness, weakness, gait disorder, falls and functional difficulties using hands, each scored using a symptom severity scale along the lines of that used in the CDR i.e. 0 (absent), 0.5 (very mild/questionable), 1 (mild), 2 (moderate), and 3 (severe) (see Supplementary Table 1 for details).

Motor features are also captured in GENFI within the structured neurological examination assessment and include face weakness, bulbar palsy, pseudobulbar palsy, neck weakness, rest tremor, postural tremor, bradykinesia, upper and lower limb weakness, upper limb apraxia, alien limb syndrome, cortical sensory loss, ataxia, abnormal gait and postural instability. In order to compare whether motor symptoms recorded in the history are compatible with motor examination deficits found on examination, we explored the relationship of each motor symptom with an approximately equivalent composite of motor examination features. This composite was given a single score of 0, 0.5, 1, 2 or 3 calculated using the same algorithm as that used to generate a CDR^®^ plus NACC FTLD global score [31]. The equivalent examination features for each symptom are shown in parentheses as follows: dysarthria (face weakness, bulbar palsy, pseudobulbar palsy), dysphagia (face weakness, bulbar palsy, pseudobulbar palsy, neck weakness), tremor (rest tremor, postural tremor); slowness (bradykinesia), weakness (face weakness, neck weakness, upper and lower limb weakness), gait disorder (ataxia, abnormal gait), falls (ataxia, abnormal gait, postural instability), and functional difficulties using hands (upper limb apraxia, alien limb syndrome, cortical sensory loss, bradykinesia and upper limb weakness).

#### Statistical analysis

All statistical analyses were performed using Stata/MP 16.1 unless otherwise stated. All graphs were produced using GraphPad Prism 9 apart from the Sankey diagrams which were made using SankeyMATIC.

Statistical tests of normality were performed using the Shapiro–Wilk test. Demographics were compared between groups using either linear regression (age and education) or a chi-squared test (sex). Linear regressions adjusting for age and sex were used to compare the MMSE and the CDR^®^ plus NACC FTLD. Individual motor symptoms were compared in each disease group versus controls using linear regressions adjusting for age and sex, and 95% bias-corrected bootstrapped confidence intervals with 2000 repetitions (as there was minimal variation from zero in severity scores for the control group), and between genetic groups using an ordinal logistic regression adjusting for age and sex.

Principal component analysis (PCA) using R version 4.1.2 [39] was performed in all mutation carriers together to identify how different motor symptoms loaded together. Components with an eigenvalue greater than one were selected and the varimax rotation was used. Similar PCA were also performed in the *C9orf72* and *GRN* mutation carriers, but insufficient nonzero scores precluded a separate PCA within the *MAPT* group.

#### Rating scale analysis

Finally, we investigated adding a motor component to the CDR^®^ plus NACC FTLD rating scale. Firstly, we used a single global score that was part of the GENFI clinical assessment where the clinician was asked to give an overall judgement of the severity of motor symptoms (called here the Global Motor Score—see Supplementary Table 1). We compared the addition of this score to the CDR^®^ plus NACC FTLD (which we call the CDR^®^ plus NACC FTLD-M) with the original CDR^®^ plus NACC FTLD. Secondly, we investigated using a different type of motor score that combines the individual motor symptoms recorded within the GENFI symptom scales (using the methodology described in Supplementary Table 2) rather than the global score. We then compared the addition of this Algorithm-based Motor Score to the CDR^®^ plus NACC FTLD (which we call the CDR^®^ plus NACC FTLD-MI, for *individual* symptoms) with the CDR^®^ plus NACC FTLD-M.

## Results

### Demographics

No significant differences were seen between the mutation groups in years of education, but the overall mutation carrier group and *C9orf72* mutation carriers had, on average, significantly fewer education years than controls (*p* = 0.024 and *p* = 0.048, respectively). *C9orf72* and *GRN* mutation carriers were significantly older than controls (both *p* < 0.001) and *MAPT* mutation carriers (*p* < 0.001 and *p* = 0.001, respectively), whilst the *C9orf72* mutation group contained more males than the *GRN* mutation group (Chi^2^ = 3.91, *p* = 0.048) (Table 1).

### Disease severity

The MMSE and CDR^®^ plus NACC FTLD Sum of Boxes scores were significantly different to controls in each genetic group (Table 1). There were no significant differences between the mutation groups apart from for the CDR^®^ plus NACC FTLD which was higher in the overall *C9orf72* and *MAPT* groups compared with the *GRN* group (*p* = 0.016 and *p* = 0.043, respectively).

### Motor diagnoses in the GENFI cohort

**Fig. 1:**
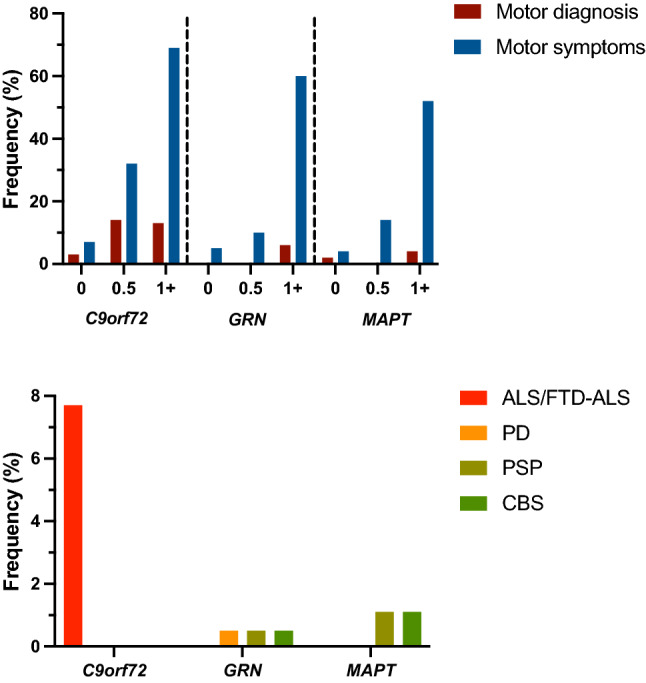
Frequency of participants with a motor diagnosis compared to those with motor symptoms, in asymptomatic (CDR^®^ plus NACC FTLD 0), prodromal (0.5) and symptomatic (≥ 1) mutation carriers. Specific motor diagnoses in the mutation carrier groups are shown below: ALS—amyotrophic lateral sclerosis, PD—Parkinson’s disease, PSP—progressive supranuclear palsy, CBS—corticobasal syndrome

In total, 4.2% of mutation carriers had a primary motor diagnosis: 3.3% with ALS or FTD-ALS and 1.0% with parkinsonism. Stratifying by individual genetic group, 7.7% of *C9orf72* mutation carriers had a motor diagnosis, all with ALS/FTD-ALS, in comparison with 1.4% of *GRN* mutation carriers, 0.5% with PSP, 0.5% with CBS and 0.5% with a diagnosis of PD, and 2.3% of *MAPT* mutation carriers, 1.1% with PSP and 1.1% with CBS. Stratifying by CDR^®^ plus NACC FTLD, 1.4% of mutation carriers with a global score of 0 (2.7% *C9orf72*, 0.0% *GRN*, 2.0% *MAPT*), 6.1% of mutation carriers with a global score of 0.5 (13.5% *C9orf72*, 0.0% *GRN*, 0.0% *MAPT*), and 8.7% of mutation carriers with a CDR^®^ plus NACC FTLD global score ≥ 1 (12.5% *C9orf72*, 5.8% *GRN*, 4.0% *MAPT*) had a motor diagnosis (Table 1, Fig. 1).

### Frequency and severity of motor symptoms in the GENFI cohort

Motor symptoms were reported in 24.3% of all mutation carriers: 31.7% of the *C9orf72* group, 18.8% of the *GRN* group, and 19.3% of the *MAPT* group. In comparison, only 6.8% of controls showed any symptoms. Stratifying by CDR^®^ plus NACC FTLD, motor symptoms occurred in 5.5% of mutation carriers with a global score of 0 (7.1% *C9orf72*, 4.6% *GRN*, 4.1% *MAPT*), 20.7% of mutation carriers with a global score of 0.5 (32.4% *C9orf72*, 9.7% *GRN*, 14.3% *MAPT*), and 63.1% of mutation carriers with a global score ≥ 1 (69.4% *C9orf72*, 59.6% *GRN*, 52.0% *MAPT*) (Table 1, Fig. 1).

In the combined mutation carrier group, slowness was the most frequent and severe motor symptom, followed by gait disorder (Table 2, Supplementary Table 3). Stratifying this group by CDR^®^ plus NACC FTLD, all of the motor symptoms were significantly more impaired than controls when the global score was ≥ 1: slowness (frequency 48.3%, mean (standard deviation) severity 0.65 (0.83)) and gait impairment (40.9%, 0.55 (0.84)) again being the most significantly impaired, but with all other symptoms being present with a frequency between 22.1 and 28.9% (Table 2) and a mean severity between 0.27 and 0.40 (Supplementary Table 3). At the prodromal stage (CDR^®^ plus NACC FTLD global score of 0.5) weakness (12.2%, 0.15 (0.51)), gait disorder (8.5%, 0.11 (0.44)) and functional difficulties using hands (8.5%, 0.12 (0.50)) were significantly impaired compared with controls. At a CDR^®^ plus NACC FTLD global score of 0 none of the symptoms were significantly impaired compared with controls.

**Table 2:** Frequency of individual motor symptoms in controls and mutation carriers. Shown as percentage of all mutation carriers in that group

	Controls	All mutation carriers			*C9orf72*			*GRN*			*MAPT*		
CDR® plus NACC FTLD		0	0.5	1 +	0	0.5	1 +	0	0.5	1 +	0	0.5	1 +
Dysarthria	1.3	0.3	7.3	26.2	0.9	13.5	31.9	0.0	3.2	26.9	0.0	0.0	8.0
Dysphagia	1.0	0.3	7.3	24.2	0.9	13.5	36.1	0.0	3.2	15.4	0.0	0.0	8.0
Tremor	4.5	3.1	4.9	26.2	3.6	2.7	33.3	3.1	6.5	25.0	2.0	7.1	8.0
Slowness	1.3	1.0	6.1	48.3	1.8	10.8	54.2	0.0	3.2	40.4	2.0	0.0	48.0
Weakness	1.3	2.4	12.2	22.1	4.5	18.9	34.7	0.8	6.5	15.4	2.0	7.1	0.0
Gait disorder	1.9	1.4	8.5	40.9	2.7	13.5	51.4	0.8	6.5	32.7	0.0	0.0	28.0
Falls	1.0	1.0	4.9	27.5	1.8	10.8	37.5	0.0	0.0	19.2	2.0	0.0	16.0
Functional difficulties using hands	0.3	2.1	8.5	28.9	2.7	16.2	37.5	1.5	3.2	23.1	2.0	0.0	16.0

Stratifying by genetic group, all symptoms were significantly impaired in the symptomatic (CDR^®^ plus NACC FTLD ≥ 1) *C9orf72* mutation carriers, with slowness and gait disorder being the most frequent (54.2%, 51.4%, respectively) and severe (0.77 (0.86), 0.71 (0.91)). Weakness (18.9%, 0.26 (0.71)) and functional difficulties using hands (16.2%, 0.26 (0.72)) were significantly impaired when CDR^®^ plus NACC FTLD was 0.5 while tremor was the least frequent and severe symptom in *C9orf72* mutation carriers (Table 2, Supplementary Table 3). In symptomatic *GRN* mutation carriers all symptoms apart from dysphagia, were significantly impaired compared with controls, with slowness and gait disorder again being the most frequent (40.4%, 32.7%) and severe (0.60 (0.88), 0.47 (0.84)). No symptoms were impaired compared with controls at a CDR^®^ plus NACC FTLD of 0 or 0.5. As with the other two groups, slowness and gait disorder were the most frequent (48.0%, 28.0%) and severe (0.40 (0.52), 0.24 (0.48)) symptoms in symptomatic *MAPT* mutation carriers (although only slowness was significantly impaired compared with controls). Other symptoms were in general less frequent, and no symptoms were significantly impaired at a CDR^®^ plus NACC FTLD of 0 or 0.5.

### Comparing motor symptoms and examination scores

In general the two different methods of measuring motor features were not substantially different: for all the mutation carriers, mean severity score was 0.12 (0.47) for motor symptom and 0.03 (0.15) for motor examination for dysarthria, 0.09 (0.36) and 0.03 (0.18) for dysphagia, 0.11 (0.37) and 0.10 (0.32) for tremor, 0.20 (0.54) and 0.13 (0.41) for slowness, 0.12 (0.44) and 0.06 (0.29) for weakness, 0.19 (0.55) and 0.08 (0.22) for gait disorder, 0.11 (0.40) and 0.12 (0.39) for falls, and 0.15 (0.49) and 0.22 (0.56) for functional difficulties using hands. On an individual basis, the symptom severity score tended to be more severe compared with the equivalent examination score for all of the motor symptoms except for falls and functional difficulties using hands where the opposite was the case (Supplementary Fig. 1).

### Principal component analysis

All motor symptoms were highly correlated with one another. Consistent with this, the motor symptoms PCA in the combined mutation carriers group loaded onto a single component, with a proportion of variance explained of 79% (Table 3). A single component was also found for the *C9orf72* and *GRN* mutation carriers. This supports the hypothesis that motor symptoms have a common underlying cause of variance, and so may be measured by a single numerical summary score.

**Table 3:** Principal component analysis of motor symptoms in all mutation carriers and in the individual genetic mutation groups

	All mutation carriers	*C9orf72*	*GRN*
Component	1	1	1
Dysarthria	0.86	0.84	0.86
Dysphagia	0.86	0.82	0.90
Tremor	0.75	0.72	0.81
Slowness	0.92	0.91	0.95
Weakness	0.89	0.89	0.88
Gait disorder	0.96	0.96	0.95
Falls	0.90	0.94	0.78
Functional difficulties using hands	0.94	0.93	0.95
Proportion of variance explained by component	0.79	0.77	0.78

### Rating scale analysis

**Fig. 2:**
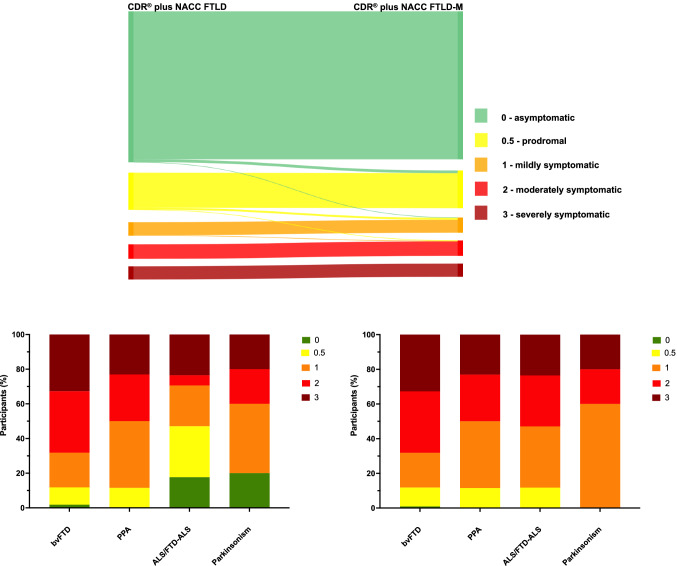
Comparison of the standard CDR^®^ plus NACC FTLD with a new CDR^®^ plus NACC FTLD plus Global Motor Score (CDR^®^ plus NACC FTLD-M). Top figure shows the change in global score in individual participants and bottom figure shows the percentage of symptomatic participants with a particular CDR score (left shows standard CDR^®^ plus NACC FTLD, right shows new CDR^®^ plus NACC FTLD-M). Diagnoses: bvFTD, behavioural variant frontotemporal dementia; PPA, Primary Progressive Aphasia; ALS/FTD-ALS, Amyotrophic Lateral Sclerosis; Parkinsonism (Progressive Supranuclear Palsy, Corticobasal Syndrome or Parkinson’s Disease)

The CDR^®^ plus NACC FTLD and CDR^®^ plus NACC FTLD-M global scores were significantly positively correlated (*ρ* = 0.980, *p* < 0.001). However, individual global scores tended to be more severe for CDR^®^ plus NACC FTLD-M: more mutation carriers were prodromal (0.5), mild (1) and moderately (2) affected with CDR^®^ plus NACC FTLD-M compared with CDR^®^ plus NACC FTLD: 16.5 versus 16.2% prodromal; 6.6% versus 5.9% mild; 6.6% versus 6.3% moderate (Fig. 2). Furthermore, no patients with ALS/FTD-ALS were classified anymore as asymptomatic (compared with 17.7% for the original CDR^®^ plus NACC FTLD), and more of this group were now classified as mildly (41.2% vs 23.5%) and moderately (17.7% vs 5.9%) affected. Similarly, no patients with parkinsonism were classified as asymptomatic anymore (compared with 20.0% for the original CDR^®^ plus NACC FTLD) (Fig. 2). No significant changes were seen in the distributions of the bvFTD and PPA groups.

The Global Motor Score was significantly positively correlated with the Algorithm-based Motor Score (*ρ* = 0.902, *p* < 0.001). However, slightly fewer people tended to be scored as asymptomatic (82.2% vs 85.6%) and more people scored as very mild (8.9% vs 5.8%) using the Algorithm-based Motor Score (Supplementary Fig. 2). Comparing each of them together as part of an addition to the CDR^®^ plus NACC FTLD, this translated into a slightly greater percentage being scored as prodromal (0.5: 16.5% for CDR^®^ plus NACC FTLD-M vs 18.2% for CDR^®^ plus NACC FTLD-MI) rather than asymptomatic (0: 64.5% vs 63.0%) for the CDR^®^ plus NACC FTLD-MI (Supplementary Fig. 3).

## Discussion

This study has shown that motor symptoms are common in genetic FTD, occurring more frequently than primary motor diagnoses, and affecting *C9orf72* more than *MAPT* or *GRN* mutation carriers. Slowness and gait impairment were the most common motor symptoms in the cohort but PCA revealed that all motor symptoms were strongly correlated and loaded together in a single component. Addition of a Global Motor Score to the CDR^®^ plus NACC FTLD scale produced a new CDR^®^ plus NACC FTLD-M scale which led to a more accurate measure of disease severity than the original scale, e.g. no patients clinically judged to be symptomatic and diagnosed with a primary motor disorder now had a global score of 0 i.e. considered asymptomatic on the CDR^®^ plus NACC FTLD scale.

Motor symptoms were present in a quarter of the cohort, much higher than the 4% of participants with a primary motor diagnosis. However, there was an increase in the number of people with motor symptoms as disease severity increased, from 6% asymptomatically to 21% in the prodromal period, to 63% at the symptomatic stage. The steep increase in frequency of motor symptoms noted when mutation carriers become symptomatic suggests that their development occurs at a later stage of genetic FTD for many people [3–5]. Nonetheless, around a third of symptomatic patients remain without any motor impairment within the GENFI cohort at time of assessment.

Overall, slowness and gait disorder were the most common of the motor symptoms, when all mutation carriers were considered together and also in each individual genetic group. This may well be because these two features both encompass a number of different neurological features. Slowness could be due to the presence of bradykinesia in a parkinsonian disorder, but also due to the presence of weakness or rigidity in a motor neuron disease. Similarly, a person’s gait can be abnormal in genetic FTD due to many reasons including both an akinetic-rigid syndrome or ALS, as well as ataxia in some cases.

Within the genetic mutation groups *C9orf72* mutation carriers had the most symptoms at every CDR stage with features mostly suggestive of ALS (including both limb and bulbar features). In contrast, *GRN* and *MAPT* mutation carriers had fewer individuals with symptoms, although in those who did have motor impairment, features were suggestive of parkinsonism (with fewer bulbar symptoms). These findings are consistent with the distribution of motor diagnoses across the genetic groups, with ALS/FTD-ALS occurring exclusively in *C9orf72* mutation carriers, and parkinsonian disorders occurring more commonly in *GRN* and *MAPT* mutation carriers. In turn, this fits in with the known literature on motor diagnoses in genetic FTD [20, 23–25, 40–43].

The focus of this study was on patient and caregiver-reported symptoms in order to be able to design a module for a clinical rating scale that was patient-focused and relevant to quality of life. However, in day-to-day clinical practice (and in the GENFI study) a physical neurological examination is performed to reinforce and extend the findings from the clinical history. Whilst these may correspond relatively well, signs can be present on examination without causing symptoms and, vice versa, some symptoms can be reported despite little to find on examination. Although it is difficult to exactly match symptoms described from the history to motor signs found on neurological examination, we performed a comparison of symptoms and signs to ensure there were no major inconsistencies between these two assessment methods. Overall, the matched motor symptom and examination scores were similar, albeit with a tendency for the symptom severity to be more severe in most of the domains. We felt this was supportive of using the GENFI symptom questionnaire as the main input to a new clinical rating scale motor module.

In the PCA all motor symptoms loaded together in mutation carriers as a whole and within both the *C9orf72* and *GRN* genetic groups (with not enough data currently to fit a separate PCA for *MAPT* mutations). This is distinct from a previous study focused on motor signs rather than symptoms in the GENFI study which found five components described as bulbar ALS-like, spinal ALS-like, PSP-like, CBS-like, and PD-like (Schonecker et al., in press). As per the discussion above, this difference is likely to be related to the lack of one-to-one correspondence between symptoms and signs, and the fact that many symptoms may encompass a number of different physical signs across ALS and parkinsonism. The finding of one component only in this study within the PCA provides support for a single motor module to be added to any clinical rating scale as long as symptoms rather than signs are considered.

The addition of a Global Motor Score to the CDR^®^ plus NACC FTLD to form the CDR^®^ plus NACC FTLD-M leads to a more accurate measure of disease severity within genetic FTD by accounting for symptoms not previously included. This is reflected in generally higher global scores in participants, i.e. increased disease severity. In particular, individuals with ALS/FTD-ALS and parkinsonism (PD, PSP, CBS) are no longer scored artificially low in their disease severity (and in some cases at a score of 0 i.e. ‘asymptomatic’ on the CDR^®^ plus NACC FTLD). However, the increase in score was not just in those with a primary motor diagnosis as the bvFTD group had higher CDR^®^ plus NACC FTLD-M scores than CDR^®^ plus NACC FTLD, consistent with the additional presence of motor features in bvFTD, as reported in multiple prior studies. In contrast, there was little change in the PPA group where motor symptoms may occur but are less frequent [44–46]. Overall, the increased accuracy of this new scale is particularly important for forthcoming clinical trials which require scales that are able to detect mutation carriers at the earliest disease stages and are able to account for the whole spectrum of phenotypes within genetic FTD.

Global Motor and Algorithm-based Motor Scores were strongly correlated but interestingly people tended to have a higher score when individual symptoms were measured and combined in the Algorithm-based Motor Score, rather than when a Global Motor Score was recorded. It may be that by focusing on individual symptoms more deficits are noted than when just asking clinicians to record an overall global score of motor dysfunction, which can be difficult to assess (and may sometimes be more of a subjective ‘feel’) given the diversity of the different features. This perhaps adds increased objectivity to the motor module in the CDR plus NACC FTLD-MI than the CDR plus NACC FTLD-M, which is important when thinking about running multicentre trials with multiple different raters, with an aim of reducing inter-rater variability.

There remain some limitations of the study. Whilst overall numbers are large for a study of genetic FTD, individual group numbers are less once stratified, and a PCA was not possible within the *MAPT* mutation group. Furthermore, the study focused on cross-sectional data and future studies should investigate change in motor features over time, and particularly how the CDR^®^ plus NACC FTLD-M performs longitudinally. Lastly, although each rater received training in use of the scales, it will be important in future studies to formally assess both intra- and inter-rater variability.

In summary, motor symptoms are a key feature of genetic FTD, with differences in the type and extent of motor impairment noted between the main genetic mutation groups. Importantly, motor symptoms occur commonly in people without a primary motor diagnosis. Hence, incorporating a motor domain into a clinical rating scale for genetic FTD is essential for future trials. This will improve disease staging which in turn should optimise not only the stratification of individuals into trials but also the accuracy of clinical outcome measures.

## Supplementary Information

Below is the link to the electronic supplementary material.

Supplementary file1 (PDF 416 KB)
