# Supplementary material for: Motor symptoms in genetic frontotemporal dementia: developing a new module for clinical rating scales
Source: J Neurol. 2022 Nov 17;270(3):1466–77. doi: 10.1007/s00415-022-11442-y (PMC9971048; doi:10.1007/s00415-022-11442-y)

Motor symptoms in genetic frontotemporal dementia – developing a new module for clinical rating scales, *Journal of Neurology*

Kiran Samra[1], Amy M. MacDougall[2], Georgia Peakman[1], Arabella Bouzigues[1], Martina Bocchetta[1], David M. Cash[1], Caroline V. Greaves[1], Rhian S. Convery[1], John C. van Swieten[3], Lize Jiskoot[3], Harro Seelaar[3], Fermin Moreno[4,5], Raquel Sanchez-Valle[6], Robert Laforce[7], Caroline Graff[8,9], Mario Masellis[10], Carmela Tartaglia[11], James B. Rowe[12], Barbara Borroni[13], Elizabeth Finger[14], Matthis Synofzik[15,16], Daniela Galimberti[17,18], Rik Vandenberghe[19,20,21], Alexandre de Mendonça[22], Chris R. Butler[23,24], Alexander Gerhard[25,26], Simon Ducharme[27,28], Isabelle Le Ber[29,30,31,32], Pietro Tiraboschi[33], Isabel Santana[34,35], Florence Pasquier[36,37,38], Johannes Levin[39,40,41], Markus Otto[42], Sandro Sorbi[43,44], Jonathan D. Rohrer[1*], Lucy L. Russell[1*], on behalf of the Genetic FTD Initiative (GENFI)[#]

*joint senior authors

[#]List of consortium authors in appendix

[1]Dementia Research Centre, Department of Neurodegenerative Disease, UCL Queen Square Institute of Neurology, London, UK.

[2]Department of Medical Statistics, London School of Hygiene and Tropical Medicine, London, UK

[3]Department of Neurology, Erasmus Medical Centre, Rotterdam, Netherlands

[4]Cognitive Disorders Unit, Department of Neurology, Donostia Universitary Hospital, San Sebastian, Spain

[5]Neuroscience Area, Biodonostia Health Research Institute, San Sebastian, Gipuzkoa, Spain

[6]Alzheimer's disease and Other Cognitive Disorders Unit, Neurology Service, Hospital Clínic, Institut d'Investigacións Biomèdiques August Pi I Sunyer, University of Barcelona, Barcelona, Spain

[7]Clinique Interdisciplinaire de Mémoire, Département des Sciences Neurologiques, CHU de Québec, and Faculté de Médecine, Université Laval, QC, Canada

[8]Center for Alzheimer Research, Division of Neurogeriatrics, Department of Neurobiology, Care Sciences and Society, Bioclinicum, Karolinska Institutet, Solna, Sweden

[9]Unit for Hereditary Dementias, Theme Aging, Karolinska University Hospital, Solna, Sweden

[10]Sunnybrook Health Sciences Centre, Sunnybrook Research Institute, University of Toronto, Toronto, Canada

[11]Tanz Centre for Research in Neurodegenerative Diseases, University of Toronto, Toronto, ON, Canada

[12]Department of Clinical Neurosciences, University of Cambridge, UK

[13]Neurology Unit, Department of Clinical and Experimental Sciences, University of Brescia, Brescia, Italy
[14]Department of Clinical Neurological Sciences, University of Western Ontario, London, ON, Canada
[15]Department of Neurodegenerative Diseases, Hertie-Institute for Clinical Brain Research and Center of Neurology, University of Tübingen, Tübingen, Germany
[16]Center for Neurodegenerative Diseases (DZNE), Tübingen, Germany
[17]Fondazione Ca' Granda, IRCCS Ospedale Policlinico, Milan, Italy
[18]University of Milan, Centro Dino Ferrari, Milan, Italy
[19]Laboratory for Cognitive Neurology, Department of Neurosciences, KU Leuven, Leuven, Belgium
[20]Neurology Service, University Hospitals Leuven, Leuven, Belgium
[21]Leuven Brain Institute, KU Leuven, Leuven, Belgium
[22]Laboratory of Neurosciences, Institute of Molecular Medicine, Faculty of Medicine, University of Lisbon, Lisbon, Portugal
[23]Nuffield Department of Clinical Neurosciences, Medical Sciences Division, University of Oxford, Oxford, UK
[24]Department of Brain Sciences, Imperial College London, UK
[25]Division of Neuroscience and Experimental Psychology, Wolfson Molecular Imaging Centre, University of Manchester, Manchester, UK
[26]Departments of Geriatric Medicine and Nuclear Medicine, University of Duisburg-Essen, Germany
[27]Department of Psychiatry, McGill University Health Centre, McGill University, Montreal, Québec, Canada
[28]McConnell Brain Imaging Centre, Montreal Neurological Institute, McGill University, Montreal, Québec, Canada
[29]Sorbonne Université, Paris Brain Institute – Institut du Cerveau – ICM, Inserm U1127, CNRS UMR 7225, AP-HP - Hôpital Pitié-Salpêtrière, Paris, France
[30]Centre de référence des démences rares ou précoces, IM2A, Département de Neurologie, AP-HP - Hôpital Pitié-Salpêtrière, Paris, France
[31]Département de Neurologie, AP-HP - Hôpital Pitié-Salpêtrière, Paris, France
[32]Reference Network for Rare Neurological Diseases (ERN-RND)
[33]Fondazione IRCCS Istituto Neurologico Carlo Besta, Milano, Italy
[34]University Hospital of Coimbra (HUC), Neurology Service, Faculty of Medicine, University of Coimbra, Coimbra, Portugal
[35]Center for Neuroscience and Cell Biology, Faculty of Medicine, University of Coimbra, Coimbra, Portugal
[36]Univ Lille, France
[37]Inserm 1172, Lille, France

[38]CHU, CNR-MAJ, Labex Distalz, LiCEND Lille, France

[39]Department of Neurology, Ludwig-Maximilians Universität München, Munich, Germany

[40]German Center for Neurodegenerative Diseases (DZNE), Munich, Germany

[41]Munich Cluster of Systems Neurology (SyNergy), Munich, Germany

[42]Department of Neurology, University of Ulm, Germany

[43]Department of Neurofarba, University of Florence, Italy

[44]IRCCS Fondazione Don Carlo Gnocchi, Florence, Italy

Corresponding author:

Dr Lucy L. Russell, Dementia Research Centre, Department of Neurodegenerative Disease, UCL Institute of Neurology, Queen Square, London, WC1N 3BG, l.russell@ucl.ac.uk

Online only (supplementary) material

Number of supplementary tables: 3
Number of supplementary figures: 3

Supplementary eTable 1 Motor symptoms included in the GENFI Symptom Scales in the History assessment. Scoring of each symptom is on a scale similar to the CDR i.e. 0 (absent), 0.5 (very mild/questionable), 1 (mild), 2 (moderate), and 3 (severe).

Supplementary eTable 2 Scoring rules to derive the algorithm-based motor score (as per the method used to calculate the CDR® plus NACC FTLD Global Score)

Supplementary eTable 3 Mean (standard deviation) severity of motor symptoms in controls and mutation carriers. Bold items are significantly different to controls using linear regression analysis (p < 0.05). Other differences are shown as [a]significantly impaired compared to *GRN* and [b]significantly impaired compared to *MAPT* using logistic regression analysis (p < 0.05).

Supplementary eFigure 1 Frequency of each individual motor symptom score within all mutation carriers compared with the equivalent motor examination score. In the left-hand figure for each motor feature, frequency is on the y-axis, and the severity of the motor symptom or examination scores is shown on the x-axis with the following scale: 0 = absent, 0.5 = very mild, 1 = mild, 2 = moderate, 3 = severe. In the right-hand figure, a Sankey diagram illustrates the difference in numbers of cases with a particular severity score, with motor symptoms on the left of each diagram, and motor examination on the right.

Supplementary eFigure 2 Comparison of the Global Motor Score and the Algorithm-based Motor Score: 0 = absent, 0.5 = very mild, 1 = mild, 2 = moderate and 3 = severe.

Supplementary eFigure 3 Comparison of the CDR® plus NACC FTLD plus Global Motor Score (CDR® plus NACC FTLD-M) with the CDR® plus NACC FTLD plus Algorithm-based Motor Score (CDR® plus NACC FTLD-MI).

Supplementary eTable 1 Motor symptoms included in the GENFI Symptom Scales in the History assessment. Scoring of each symptom is on a scale similar to the CDR i.e. 0 (absent), 0.5 (very mild/questionable), 1 (mild), 2 (moderate), and 3 (severe).

	Questionable/Very mild	Mild	Moderate	Severe
**Global Motor Score**	Questionable/very mild motor impairment.	Mild motor impairment.	Moderate motor impairment.	Severe motor impairment.
**Dysarthria**<br>Has the subject had difficulties with articulation?	Possible speech disturbance of questionable significance or very mild.	Detectable speech disturbance – may be asked to repeat statements infrequently.	Becoming less intelligible – frequently asked to repeat statements.	Generally unintelligible.
**Dysphagia**<br>Has the subject had difficulties with swallowing?	Rare choking of questionable significance.	Swallowing problems are evident with regular episodes of choking.	Requires changes in dietary consistency.	Needs supplemental tube feeding.
**Tremor**<br>Has the subject had rhythmic shaking, especially in the hands, arms, legs or head?	Tremor is infrequently present and of questionable significance or very mild.	Tremor is evident but is generally not distressing to the patient.	Tremor is evident, may be distressing and interferes with many activities.	Tremor interferes with most activities.
**Slowness**<br>Has the subject noticeably slowed down in walking or moving or handwriting, other than injury or illness? Has the subject's facial expression changed?	Slight slowing down of movements of questionable significance or very mild.	Mild slowing – slower walking but requires little or no assistance; writing slower but still legible; definite diminution of facial expression.	Moderate slowing – walks with assistance; not all words legible when writing; lips parted some of the time.	Severe slowing – cannot walk; majority of words not legible; severe or complete loss of facial expression.
**Weakness**<br>Has the subject noticed their arms or legs have become weak?	Possible weakness of limbs but of questionable significance or very mild.	Mild weakness – does not require assistance with using arms or walking.	Moderate weakness – requires assistance walking or with activities requiring the arms.	Severe weakness – unable to walk and/or use arms.
**Gait disorder**<br>Has the subject's walking changed, not specifically due to arthritis or injury? Is the subject unsteady, or shuffle when walking, or drag a foot?	Possibly some difficulties with walking but of questionable significance or very mild.	Requires little or no assistance.	Walks with assistance.	Cannot walk at all, even with assistance.
**Falls**<br>Does the subject fall more than usual?	Rare falls of questionable significance.	Occasional falls but less than once per day.	Falls on average once daily.	Falls more than once daily.
**Functional difficulties using hands**<br>E.g. using knife and fork, buttoning clothes, washing hands and face	Possible functional difficulties using hands but of questionable significance or very mild.	Requires little or no assistance.	Requires assistance.	Cannot use hands at all even with assistance.

Supplementary eTable 2 Scoring rules to derive the algorithm-based motor score (as per the method used to calculate the CDR® plus NACC FTLD Global Score)

Individual scores	Overall score
All 0	0
Maximum 0.5	0.5
Maximum > 0.5:	
Maximum 1, all others 0	0.5
Maximum 2 or 3, all others 0	1
Maximum occurs once, another rating > 0	One level < maximum
Maximum occurs > once	Maximum score

Supplementary eTable 3 Mean (standard deviation) severity of motor symptoms in controls and mutation carriers. Bold items are significantly different to controls using linear regression analysis ($p < 0.05$). Other differences are shown as [a]significantly impaired compared to *GRN* and [b]significantly impaired compared to *MAPT* using logistic regression analysis ($p < 0.05$).

	Controls	All mutation carriers			*C9orf72*			*GRN*			*MAPT*		
**CDR® plus NACC FTLD**		**0**	**0.5**	**1+**	**0**	**0.5**	**1+**	**0**	**0.5**	**1+**	**0**	**0.5**	**1+**
**Dysarthria**	0.01 (0.06)	0.01 (0.12)	0.08 (0.37)	**0.37 (0.79)**	0.02 (0.19)	0.16 (0.54)	**0.40 (0.78)**[b]	0.00 (0.00)	0.02 (0.09)	**0.45 (0.95)**	0.00 (0.00)	0.00 (0.00)	0.08 (0.28)
**Dysphagia**	0.01 (0.07)	0.00 (0.03)	0.08 (0.37)	**0.27 (0.58)**	0.00 (0.05)	0.16 (0.54)	**0.39 (0.66)**[ab]	0.00 (0.00)	0.02 (0.09)	0.19 (0.55)	0.00 (0.00)	0.00 (0.00)	0.06 (0.22)
**Tremor**	0.03 (0.14)	0.02 (0.13)	0.04 (0.17)	**0.31 (0.62)**	0.03 (0.15)	0.01 (0.08)	**0.40 (0.71)**[b]	0.02 (0.14)	0.05 (0.20)	**0.29 (0.59)**	0.01 (0.07)	0.07 (0.27)	0.08 (0.28)
**Slowness**	0.01 (0.09)	0.01 (0.14)	0.04 (0.18)	**0.65 (0.83)**	0.03 (0.21)	0.07 (0.21)	**0.77 (0.86)**	0.00 (0.00)	0.03 (0.18)	**0.60 (0.88)**	0.02 (0.14)	0.00 (0.00)	**0.40 (0.52)**
**Weakness**	0.01 (0.10)	0.03 (0.22)	**0.15 (0.51)**	**0.27 (0.62)**	0.06 (0.30)	**0.26 (0.71)**[a]	**0.39 (0.69)**[a]	0.00 (0.04)	0.05 (0.20)	**0.22 (0.63)**	0.04 (0.29)	0.07 (0.27)	0.00 (0.00)
**Gait disorder**	0.01 (0.07)	0.02 (0.20)	**0.11 (0.44)**	**0.55 (0.84)**	0.04 (0.27)	0.19 (0.60)	**0.71 (0.91)**[a]	0.02 (0.18)	0.06 (0.25)	**0.47 (0.84)**	0.00 (0.00)	0.00 (0.00)	0.24 (0.48)
**Falls**	0.01 (0.07)	0.01 (0.14)	0.06 (0.35)	**0.32 (0.63)**	0.03 (0.21)	0.14 (0.52)	**0.45 (0.70)**[a]	0.00 (0.00)	0.00 (0.00)	**0.23 (0.60)**	0.02 (0.14)	0.00 (0.00)	0.14 (0.34)
**Functional difficulties using hands**	0.00 (0.06)	0.02 (0.18)	**0.12 (0.50)**	**0.40 (0.74)**	0.03 (0.22)	**0.26 (0.72)**[a]	**0.55 (0.83)**	0.01 (0.06)	0.02 (0.09)	**0.31 (0.67)**	0.04 (0.29)	0.00 (0.00)	0.16 (0.45)

Supplementary eFigure 1 Frequency of each individual motor symptom score within all mutation carriers compared with the equivalent motor examination score. In the left-hand figure for each motor feature, frequency is on the y-axis, and the severity of the motor symptom or examination scores is shown on the x-axis with the following scale: 0 = absent, 0.5 = very mild, 1 = mild, 2 = moderate, 3 = severe. In the right-hand figure, a Sankey diagram illustrates the difference in numbers of cases with a particular severity score, with motor symptoms on the left of each diagram, and motor examination on the right.

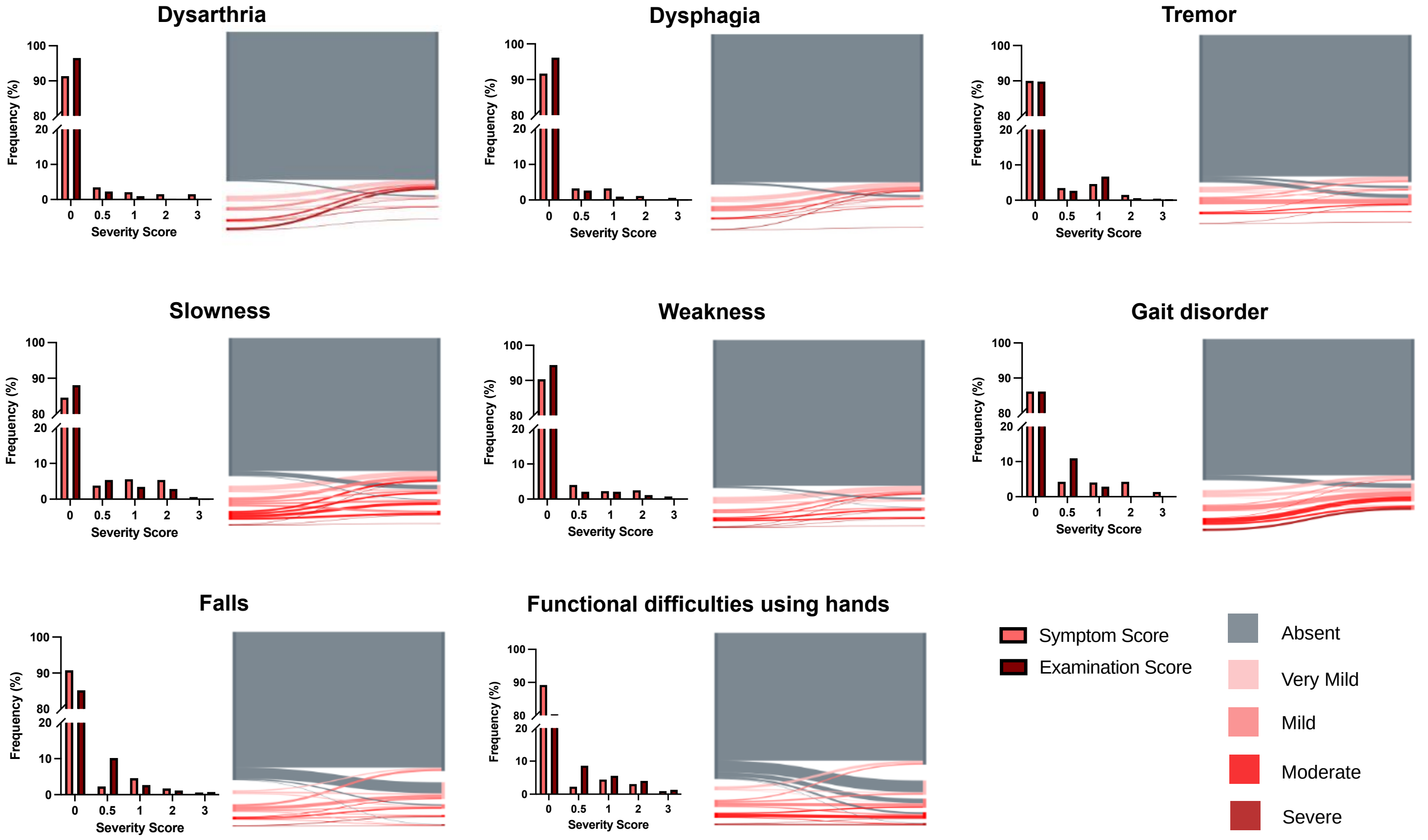

Supplementary eFigure 2 Comparison of the Global Motor Score and the Algorithm-based Motor Score: 0 = absent, 0.5 = very mild, 1 = mild, 2 = moderate and 3 = severe.

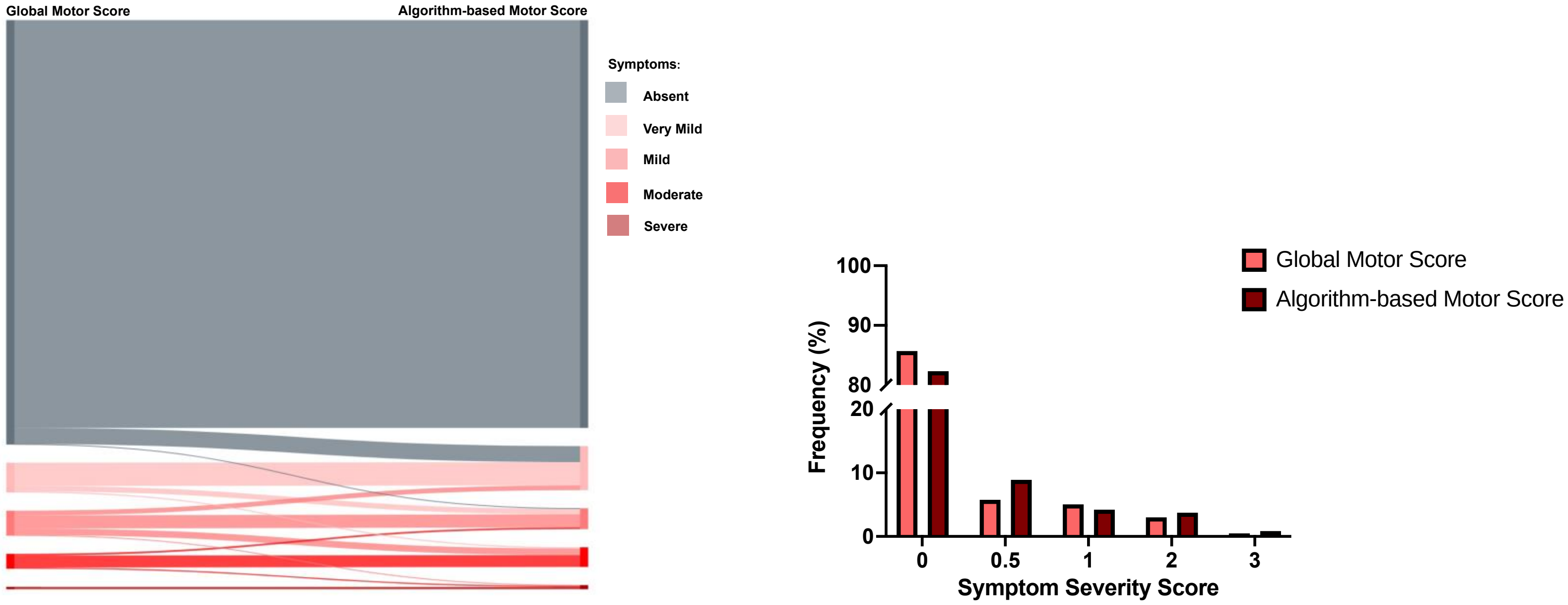

Supplementary eFigure 3 Comparison of the CDR® plus NACC FTLD plus Global Motor Score (CDR® plus NACC FTLD-M) with the CDR® plus NACC FTLD plus Algorithm-based Motor Score (CDR® plus NACC FTLD-MI).

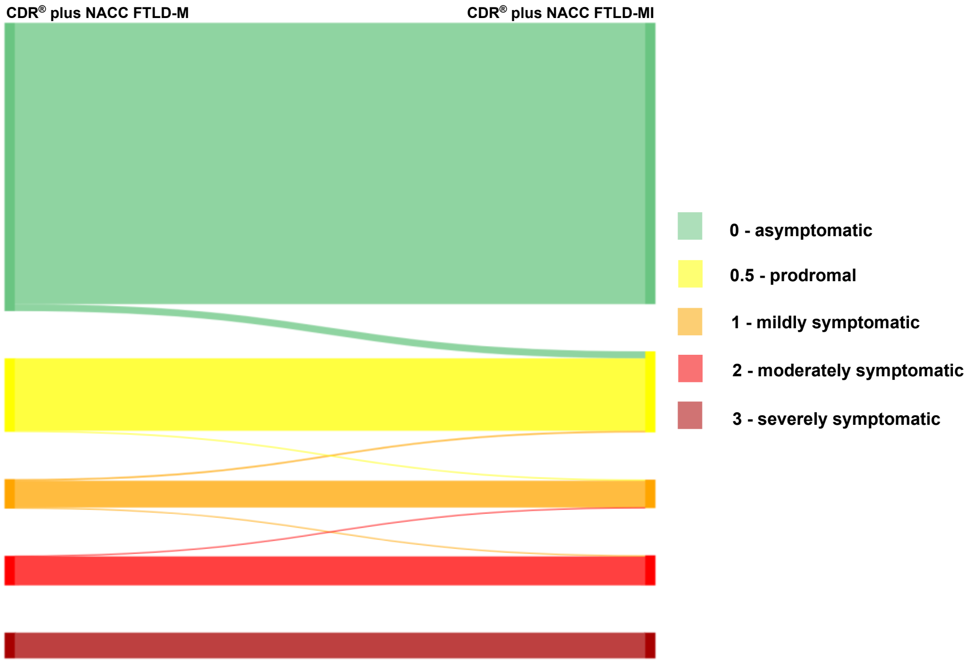

Supplement: Supplementary file 1 — Supplementary file1 (PDF 416 KB) [file 415_2022_11442_MOESM1_ESM.pdf]
